# A case of primary lung cancer requiring differentiation from metastatic cervical cancer

**DOI:** 10.1002/rcr2.1196

**Published:** 2023-07-31

**Authors:** Yoko Kataoka, Takuya Fujita, Kentaro Fukunaga, Jun Hanaoka

**Affiliations:** ^1^ Department of Surgery Shiga University of Medical Science Otsu Japan; ^2^ Department of General Thoracic Surgery Kohka Public Hospital Kohka Japan; ^3^ Department of Respiratory Medicine Kohka Public Hospital Kohka Japan

**Keywords:** HPV, lung cancer, p16, papillomavirus, squamous cell carcinoma

## Abstract

p16 has been used as a surrogate marker for human papillomavirus (HPV)‐related tumours. However, it remains unclear whether p16 is also a potential marker for pulmonary tumours. Herein, we report the case of an 80‐year‐old woman with a history of papillary squamous cell carcinoma of the uterine cervix, presenting with a left pulmonary tumour. A bronchoscopic biopsy revealed squamous cell carcinoma with a papillary pattern, which did not rule out pulmonary metastasis from the cervix. Immunohistochemical staining revealed that the cervical tumour was positive for p16, whereas the pulmonary tumour was negative and was effectively diagnosed as primary pulmonary carcinoma.

## Graphical abstract

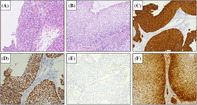

## INTRODUCTION

In patients with locally advanced squamous cell carcinoma of the cervix (SCCC), lung metastases may be observed as a recurrence. In patients with SCCC history and diagnosis of squamous cell carcinoma (SCC) of the lung, it is important to distinguish between primary lung cancer and potential metastases from SCCC to select the appropriate treatment. Human papillomavirus (HPV) infection is the leading cause of carcinogenesis in a majority of SCC of the uterine cervix.^1^, ^2^ HPV integrates into the chromosome and destabilizes it, producing oncoproteins E6 and E7, which bind to the tumour suppressor gene RB, releasing the transcription factor E2F and inducing p16 expression.^3^ Therefore, p16 has been reported to be useful as a surrogate marker to assess the status of HPV infection in SCCC and that of the head and neck.^4^, ^5^ However, the significance of p16 expression in primary lung cancer remains to be elucidated. Herein, we report a case of primary squamous cell lung cancer, which was effectively diagnosed using p16 immunohistochemistry to differentiate lung metastasis from the primary SCCC.

## CASE REPORT

**FIGURE 1:**
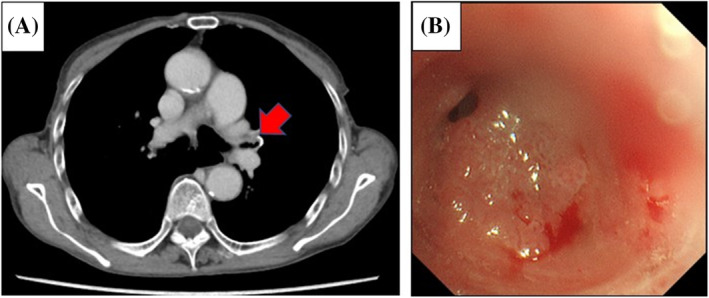
(A) Contrasted‐enhanced computed tomography (CT) revealed a 10 mm nodule in the left pulmonary hilum (arrow). (B) Bronchoscopy revealed a poor‐circumscribed tumour located at the left upper lobe bronchus.

**FIGURE 2:**
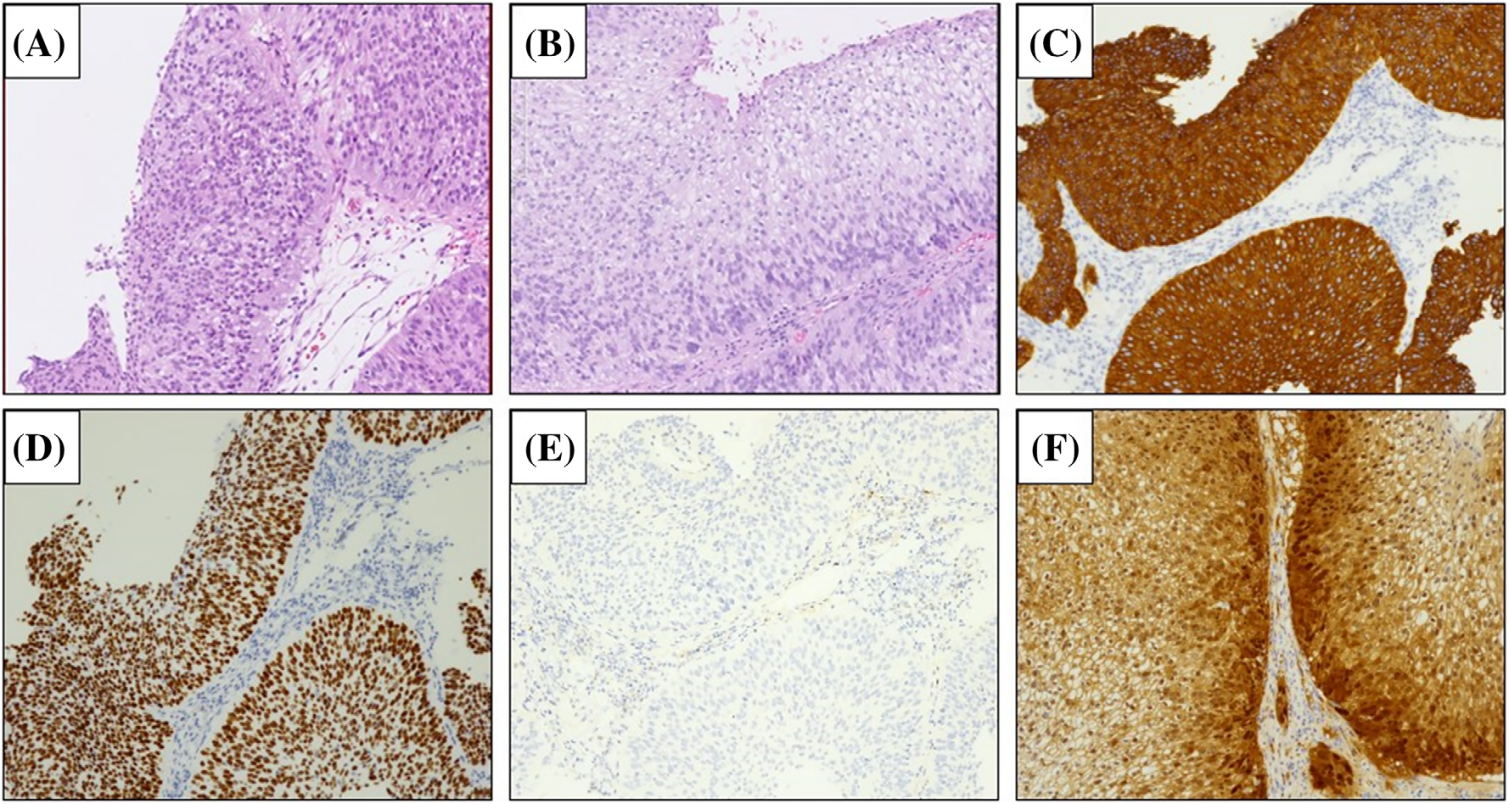
(A) Photomicrograph of haematoxylin and eosin staining of the lung tumour section showing consistency with neoplastic cells in papillary pattern (amplification ×200). (B) Photomicrograph of haematoxylin and eosin staining of the cervical tumour section revealing papillary growth of atypical neoplastic squamous cells with partially pleomorphic nuclei (amplification ×200). (C, D) Photomicrographs of immunostained lung tumour sections showing positive results for CK5/6 (C) and p40 markers (D) (amplification ×100). (E, F) Photomicrographs of immunostained lung tumour sections showing p16‐negative expression in lung tumour cells (E) and p16‐positive expression in the tumour cells of cervix cancer (F) (amplification ×200).

An 80‐year‐old asymptomatic woman was referred to our hospital after abnormal findings were identified during routine computed tomography (CT). The patient was in the 2‐year follow‐up period for papillary SCCC, which was previously treated with chemoradiotherapy. She had a history of a 30‐pack‐year smoking habit, without any presenting respiratory symptoms. Contrasted‐enhanced CT showed a 10‐mm‐nodule in the left pulmonary hilum (Figure 1A). No local or regional recurrence of SCCC was observed. The only increased tumour marker was the SCC antigen (3.4 ng/mL). Bronchoscopy revealed a poorly circumscribed tumour with edema and angiogenesis in the left upper lobe bronchus (Figure 1B), almost completely obstructing the bronchial lumen. A histological examination suggested that the lung tumour was composed of a solid sheet and displayed a papillary pattern (Figure 2A). Bronchoscopic findings suggested primary lung SCC, but the papillary pattern in the lung tumour resembled that of the previously treated SCCC (Figure 2B), which did not rule out lung metastasis. After immunohistochemical staining, the lung tumour cells were positive for CK5/6 and p40 markers (Figure 2C, D) and negative for TTF‐1, CK7 and p16 (Figure 2E). Meanwhile, the cervical carcinoma cells from the previous biopsy were strongly positive for p16 expression (Figure 2F). Conclusively, the morphologies of the lung tumour and cervical cancer cells were similar but differed in terms of p16 expression. The lung tumour was finally diagnosed as primary lung SCC. Surgery could not be performed because of a poor performance status (PS) and compromised pulmonary function. The patient underwent radiotherapy for lung cancer and was well for 14 months after treatment, with no evidence of recurrence.

## DISCUSSION

In our case, the diagnosis of primary pulmonary carcinoma was finally reached as a result of immunohistochemical analysis, which revealed that p16 was strongly expressed in the cervical carcinoma tissue but not in the lung tumour. Radiation therapy was chosen for our patient due to poor PS and compromised pulmonary function, irrespective of whether it was primary lung SCC or lung metastasis from SCCC. However, if the patient had a good PS and pulmonary function, left upper lobectomy with bronchoplasty was considered as curative treatment for primary lung cancer, while chemotherapy or a combination of chemotherapy and surgery was selected for lung metastasis from primary SCCC. Accurate diagnosis is crucial to determine the appropriate treatment, as there are distinct treatment options for primary lung cancer and lung metastasis from primary SCCC.

Immunohistochemistry of p16 is recognized as a helpful marker in HPV‐related tumours. Despite this, p16 is overexpressed not only by HPV E7‐induced pRB inactivation but also by other factors, including mutations in cell cycle regulators CDK4 and CDK6, inflammation, ageing and stress.^6^, ^7^ Several studies have shown that p16 is overexpressed in patients with non‐small cell lung cancer.^8^, ^9^, ^10^ In cervical SCC, p16 is uniformly strongly and diffusely expressed in tumour cells,^4^ whereas in non‐small cell lung cancer, p16 expression is uneven and variable.^8^, ^9^, ^10^ Notably, the association between p16‐positive lung cancer and HPV infection remains unclear. The prevalence of HPV infection in lung cancer has been previously documented along with its incidence rate, which widely ranges from 0% to 80% (with an average of 24.5%).^11^ Several factors, including regional differences, histological types, artefacts in the evaluated samples caused by procedures (frozen, formalin‐fixed and paraffin‐embedded), and the detection method used (polymerase chain reaction, in situ hybridization and immunohistochemistry) may explain this variation.^10^, ^11^, ^12^ Lin et al. reported that 13.7% of patients (57 out of 415 patients) with primary pulmonary SCC tested positive for p16 expression, whereas HPV DNA detection was negative in p16‐positive cases.^10^ In contrast, another study reported 21 patients with a history of SCCC who developed squamous cell lung cancer and tested positive for p16 expression and HPV DNA in all cases.^12^ In addition, Lin et al. and van Boerdonk et al. have suggested that the presence of HPV DNA in lung tumour tissues does not precisely prove the contribution of HPV in the development of primary lung tumours. Interestingly, the oncoprotein HPV E6/E7 gene expression could be the most accurate evaluation for such cases.^10^, ^11^, ^12^ Therefore, these studies show remarkably low evidence of the involvement of HPV in primary lung cancer.

In our study, primary squamous cell lung cancer was diagnosable by the immunohistochemical analysis of p16. Moreover, this technique may be considered, especially in cases with prior SCCC and subsequent lung squamous cell carcinoma, as a simple and inexpensive initial test for differentiation. If p16 expression is positive in SCCC and negative in the lung tumour, as in our case, the lesion can be easily diagnosed as a primary pulmonary SCC. However, similar to that in SCCC, when p16 expression is uniformly strong and diffuse in a lung tumour, and primary or metastatic lung tumours cannot be discriminated based on the clinical course, imaging and pathological features, the presence of HPV E6/E7 oncoprotein should be evaluated.

## AUTHOR CONTRIBUTIONS

Yoko Kataoka was a major contributor in writing the manuscript. All other authors contributed to data collection and interpretation and critically reviewed the manuscript. All authors read and approved the final manuscript.

## CONFLICT OF INTEREST STATEMENT

None declared.

## ETHICS STATEMENT

The authors declare that appropriate written informed consent was obtained for the publication of this manuscript and accompanying images.

## Data Availability

The data that support the findings of this study are available on request from the corresponding author. The data are not publicly available due to privacy or ethical restrictions.
